# Atrial granules as acidic calcium stores in cardiomyocytes

**DOI:** 10.1093/ehjopen/oeaf083

**Published:** 2025-07-04

**Authors:** Emily Akerman, Daniel Aston, Eva A Rog-Zielinska, Barry Boland, Ulrich Schotten, Sander Verheule, Rebecca A Capel, Rebecca A B Burton

**Affiliations:** Department of Pharmacology, University of Oxford, OX1 3QT Oxford, UK; Department of Pharmacology, University of Oxford, OX1 3QT Oxford, UK; Institute for Experimental Cardiovascular Medicine, University Heart Center Freiburg-Bad Krozingen, and Faculty of Medicine, University of Freiburg, 79106 Freiburg im Breisgau, Germany; Department of Pharmacology and Therapeutics, University College Cork, T12 K8AF, Ireland; Departments of Physiology and Cardiology, Cardiovascular Research Institute Maastricht, Maastricht University, Maastricht 6211 LK, The Netherlands; Departments of Physiology and Cardiology, Cardiovascular Research Institute Maastricht, Maastricht University, Maastricht 6211 LK, The Netherlands; Department of Pharmacology, University of Oxford, OX1 3QT Oxford, UK; Department of Pharmacology, University of Oxford, OX1 3QT Oxford, UK; Department of Pharmacology and Therapeutics, Institute of Systems, Molecular and Integrative Biology, University of Liverpool, L69 3GE Liverpool, UK; Liverpool Centre for Cardiovascular Science, University of Liverpool and Liverpool Heart & Chest Hospital, Liverpool, UK

**Keywords:** Atrial granules, Cardiomyocytes, PAM, Atrial fibrillation, Electron microscopy

## Abstract

Acidic calcium stores significantly influence basal calcium transient amplitude and β-adrenergic responses in cardiomyocytes. Atrial myocytes contain atrial granules (AGs), small acidic organelles that store and secrete atrial natriuretic peptide (ANP) and are absent in healthy ventricular myocytes. AGs are known to be acidic and calcium-rich, but their number and location relative to other signalling sites remain unexplored. Labelling of acidic organelles in adult guinea pig cardiomyocytes showed the presence of acidic puncta throughout the cytosol. Atrial myocytes exhibited an increased concentration of acidic organelles at the nuclear poles. Live cell fluorescent studies using 4-phenyl-3-butenoic acid (PBA) to inhibit peptidylglycine α-amidating monooxygenase, a crucial component of AGs membranes, effectively eliminated staining at the nuclear poles and most acidic puncta in atrial cells, but not in ventricular cells. Our immunofluorescent labelling also emphasizes the differences in acidic punctae between atrial and ventricular myocytes by showing minimal co-localization between AG-specific ANP and lysosomal-associated membrane protein. Electron microscopy studies on goat atrial fibrillation (AF) and sham control tissue allowed visualization of AGs. Quantitative analysis revealed that AGs were positioned significantly further away from the nearest sarcoplasmic reticulum and were closer to mitochondria in AF compared to sinus rhythm control tissue. We raise the question whether the positioning of AGs is strategic for communication with other calcium-containing organelles.

## Introduction

In addition to their degradative role in cells, acidic organelles (namely lysosomes) significantly contribute to calcium signalling, influencing basal calcium transient amplitude and β-adrenergic responses in both atrial^1^ and ventricular myocytes.^2^ Lysosomes in ventricular myocytes are located near the sarcoplasmic reticulum (SR) and mitochondria, creating membrane contact sites that can potentially function as signalling microdomains.^3^ Atrial granules (AGs) are lysosome-related organelles, which secrete atrial natriuretic peptide (ANP), are known to be acidic and contain a high calcium content.^4^ The discovery of AGs and natriuretic peptides within them led to the atrium being identified as an endocrine organ.^5^ Peptidylglycine α-amidating monooxygenase,^6^ the primary membrane protein in AGs, plays a crucial role for amidated peptide biosynthesis in other cells types.^6^ However, neither ANP nor brain natriuretic peptide (BNP) is amidated, suggesting that PAM performs a non-catalytic, structural role in AGs.^7^ These actions likely are confined to the early stages of the secretory pathway.^8^ Additionally, an accumulation of AGs around the Golgi complex has been observed in rat atria.^4^ However, their number relative to lysosomes, as well as their positioning in relation to other calcium signalling sites has not been fully explored in atrial physiology and pathophysiology. Therefore, we hypothesize that AGs constitute a significant portion of acidic calcium stores in atrial cardiomyocytes and form distinct signalling microdomains with the SR and mitochondria, with these interactions potentially altered in conditions like atrial fibrillation (AF).

## Methods

### Myocyte isolation

All animal experiments were performed in accordance with the UK Home Office Guide on the Operation of Animal (Scientific Procedures) Act of 1986. Live cell imaging experiments were conducted on guinea pig isolated adult atrial and ventricular cardiomyocytes. Cell isolation methods were previously published in Capel *et al*.^2^ Briefly, guinea pig hearts were dissected and washed in heparin-containing physiological saline solution (in mM: NaCl 125, NaHCO_3_ 25, KCl 5.4, NaH_2_PO_4_ 1.2, MgCl_2_ 1, glucose 5.5, CaCl_2_ 1.8, pH to 7.4 with NaOH, 20 IU heparin per mL to prevent blood clots forming) and mounted on a Langendorff setup for retrograde perfusion via the aorta. The heart was perfused in a modified Tyrode solution containing (in mM: NaCl 136, KCl 5.4, NaHCO_3_ 12, sodium pyruvate 1, NaH_2_PO_4_ 1, MgCl_2_ 1, EGTA 0.04, and glucose 5; gassed with 95% O_2_/5% CO_2_ to maintain a pH of 7.4 at 37°C for 3 min). Solution was switched to a digestion solution: the modified Tyrode above containing 100 µM CaCl_2_ and 0.04 mg/mL Liberase™ (Roche, Penzberg, Germany) and no EGTA. After 25 min of enzymatic digestion, the heart was removed. Atria were separated from ventricles in a dissection bath. Tissue was cut into small pieces (around 2 × 2 mm^3^) followed by 1 min of gentle trituration. Atrial myocytes were stored in a high potassium medium (in mM: KCl 70, MgCl_2_ 5, K^+^ glutamine 5, taurine 20, EGTA 0.04, succinic acid 5, KH_2_PO_4_ 20, HEPES 5, glucose 10; pH to 7.2 with KOH), and ventricular myocytes were stored in modified Tyrode solution. Myocytes were left at 4°C to rest for 30 min before recordings and were used up to 4 h post isolation.

### Fluorescent immunocytochemistry and LysoTracker™ labelling

Immunolabelling and analysis were carried out using the published methods.^9^ Goat anti-ANP (AF3366) and rabbit anti-LAMP2 (PA1-655) primary antibodies were purchased commercially and used at a dilution of 1:100. Briefly, isolated guinea pig atrial myocytes were fixed in 4% paraformaldehyde–phosphate buffered saline (PBS) for 15 min and washed in PBS (three changes, 5 min each). Cells were permeabilized and blocked using the detergent Triton X-100 (0.1%), 10% horse serum, and 10% BSA in PBS (Sigma-Aldrich) for 60 min at room temperature and incubated with primary antibodies at 4°C overnight dissolved in blocking solution. Cells were first washed with PBS (three changes, 5 min each) before being incubated with secondary antibodies, AlexaFluor −488 (ANP) or −546 (LAMP2) conjugated secondary antibodies (Invitrogen, UK), raised against the appropriate species, at room temperature for 120 min in PBS then washed with PBS (three changes, 5 min each). Cells were mounted using Vectashield™.

LysoTracker™ staining of acidic organelles (Thermo Fisher, LysoTracker™ Green DND-26 L7526, 100 nM, 20 min) in isolated guinea pig myocytes was performed to analyse acidic punctate organelles throughout the cytoplasm. LysoTracker™ is an aromatic hydrophobic compound and works by selectively accumulating in acidic vesicular compartments. LysoTracker™ is able to accept protons, and the protonated probe can become trapped inside of acidic organelles and emit fluorescence upon excitation. We used 4-phenyl-3-butenoic acid (PBA, Sigma, 200 µM,^10^ 2 h) which is recognized as a substrate by PAM and which can form a covalent adduct with PAM leading to irreversible inactivation of PAM. The IC50 of PBA is 54 μM (HDAC2) and 87 μM (HDAC6).^11^ In the present study, we have used the concentration of 200 µM. This relatively high dose was chosen based on the reports of low membrane permeability for PBA, which results in poor cellular uptake and could limit its effects on AGs in atrial cardiomyocytes if used at lower concentrations. 4-Phenyl-3-butenoic acid has previously been used to investigate its effects on ciliogenesis mechanism *in vitro* at 200 µM, with the samples pre-treated for 3 h to ensure that PAM is enzymatically inactive.^10^ This mechanism of PBA-induced PAM inactivation has been shown to influence storage and secretion of ANP and BNP in atrial myocytes^7^ and to induce perturbation of AG formation in atrial and ventricular myocytes.

### Live cell spinning disc confocal studies

Cells were visualized using a Zeiss Axiovert 200 with attached Nipkow spinning disk confocal unit (CSU-10, Yokogawa Electric Corporation, Japan), and excitation light, transmitted through the CSU-10, was provided by a 488 nm diode laser (Vortran Laser Technology Inc., Sacramento, CA). Emitted light was passed through the CSU-10 and collected by an Andor iXON897 EM-CCD camera (Oxford Instruments, UK).

### High-resolution three-dimensional electron tomography

We used 3D electron tomography imaging data acquired as part of a previous study.^9^ The goat model in this study (female *Capra hircus*) is described in Ayagama *et al.*,^12^ and AF was induced and maintained in female goats (*C. hircus*) for 6 months. Goats were anaesthetized and euthanized following an open chest experiment (*n* = 4 AF goats and *n* = 4 sham control goats). Atrial goat biopsies were chemically fixed with Karnovsky’s fixative. Semi-thick (300 nm) sections were prepared and imaged at the Electron Microscopy Core Facility, European Molecular Biology Laboratory, Heidelberg, using 300 kV Tecnai TF30 (FEI Company, now Thermo Fisher Scientific, Eindhoven, the Netherlands) as described previously.^13^ Dual-axis tilt series were aligned, reconstructed, and combined using IMOD. Analysis and measurements were made using IMOD and ImageJ software. The study was carried out in accordance with the principles of the Basel declaration and regulations of European directive 2010/63/EU, and the local ethical board for animal experimentation of the Maastricht University approved the protocol.

### Statistics and analysis parameters

**Figure 1:**
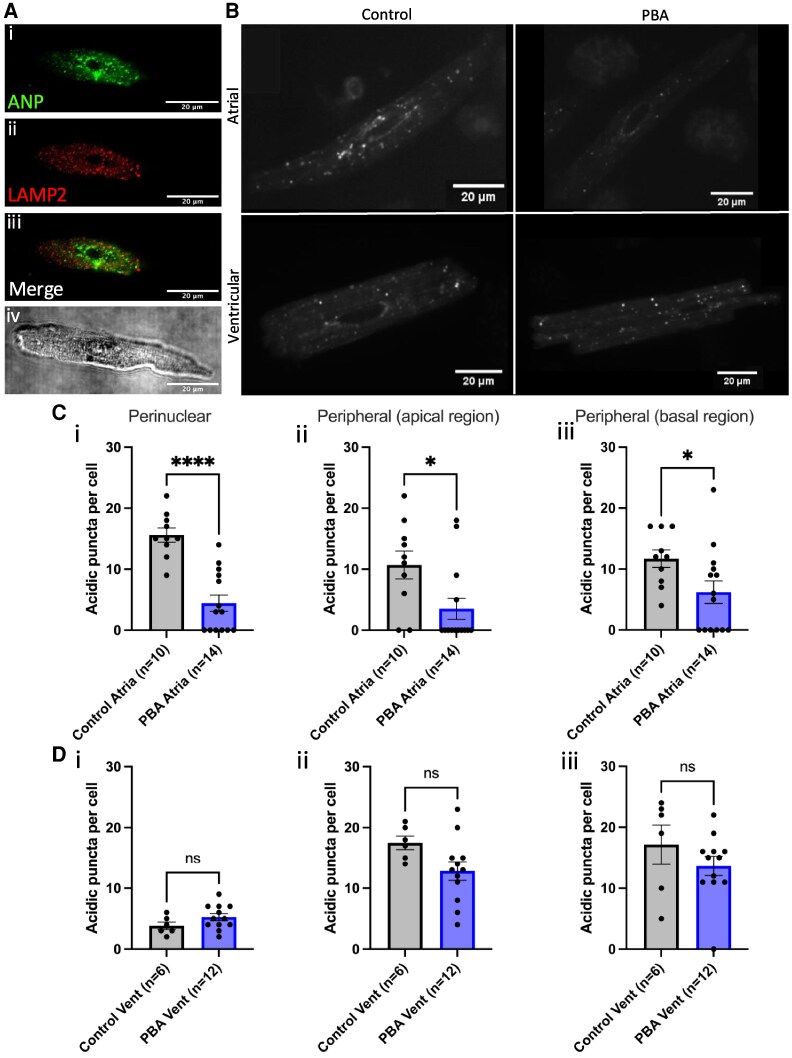
Atrial granules comprise the majority of acidic organelles in live atrial myocytes. (*A*) Representative example of a fixed, isolated guinea pig atrial myocyte immunolabelled for (i) atrial natriuretic peptide (green), (ii) lysosomal-associated membrane protein 2 (red), and (iii) co-immunolabelled for atrial natriuretic peptide and lysosomal-associated membrane protein 2; brightfield image is shown in (iv). (*B*) LysoTracker™ staining of acidic organelles (100 nM, 20 min) in primary isolated guinea pig atrial myocytes in control conditions (i) and in the presence of 4-phenyl-3-butenoic acid (200 µM, 2 h; ii) and in ventricular myocytes in control conditions (iii) and in the presence of 4-phenyl-3-butenoic acid (200 µM, 2 h; iv). (*C*) (i) Acidic perinuclear puncta count in control conditions (vehicle control *n* = 10) and in the presence of 4-phenyl-3-butenoic acid (200 µM, *n* = 14) in atrial myocytes; (ii) acidic peripheral puncta count in control conditions (vehicle control, *n* = 10) and 4-phenyl-3-butenoic acid (200 µM, *n* = 14) at the subsarcolemmal-apical region (top) of atrial myocytes; (iii) acidic peripheral puncta count in control conditions (vehicle control, *n* = 10) and 4-phenyl-3-butenoic acid (200 µM, *n* = 14) at the subsarcolemmal-basal region (bottom) of atrial myocytes. (*D*) (i) Acidic perinuclear puncta count in control conditions (vehicle control, *n* = 6) and 4-phenyl-3-butenoic acid (200 µM, *n* = 12) in ventricular myocytes; (ii) acidic peripheral puncta count in control conditions (vehicle control, *n* = 6) and 4-phenyl-3-butenoic acid (200 µM, *n* = 12) at the subsarcolemmal-apical region (top) of ventricular myocytes; (iii) acidic peripheral puncta count in control conditions (vehicle control, *n* = 6) and 4-phenyl-3-butenoic acid (200 µM, *n* = 12) at the subsarcolemmal-basal region (bottom) of ventricular myocytes. PBA, 4-phenyl-3-butenoic acid. Scale bar represents 20 μm.

Pearson’s coefficient was calculated using the JaCoP plugin in ImageJ. Background subtraction was first performed using the Rolling Ball algorithm with a radius of 10 pixels to ensure background noise was removed without losing puncta. Co-localization analysis was then conducted on eight atrial myocytes twice in two distinct areas (i) in the 5 µm radius surrounding the nucleus and (ii) in the remaining cytosol of the cell. In *Figure 1C* and *D*, the count of acidic puncta was measured using ImageJ (Fiji) software in three areas: the perinuclear zone and the peripheral zones: the subsarcolemmal-apical region (top) and the subsarcolemmal-basal region (bottom) extremities of the myocyte. The perinuclear zone was defined as a region extending 5 µm from the nuclear envelope.^14^ For the peripheral zones, imaging started from the plasmalemma and extended into the cell. For each acquisition, 25 images were taken at a frame rate of 3 frames per second. *Figure 1C* and *D* represent the frame with the highest number of puncta counted. Data sets were analysed using non-parametric Mann–Whitney *U* test. Data sets are presented as mean ± standard error of the mean. Differences were considered statistically significant at values of *P* < 0.05 and indicated as *P < 0.05, **P < 0.01, ***P < 0.001, and ****P < 0.0001. Statistical analyses were performed using Prism 10 (GraphPad, CA, USA).

## Results

### Atrial granules comprise the majority of acidic organelles in live atrial myocytes

*Figure 1A* shows confocal images of atrial myocytes labelled with primary antibodies against ANP (i, ANP) and lysosomal-associated membrane protein (ii, LAMP2), a protein localized to lysosomal membranes. Atrial natriuretic peptide exhibited expected punctate staining with low co-localization with LAMP2 puncta in the cytosol [*Figure 1A* (iii)] and significantly lower co-localization in the 5 µm radius from the nuclear envelope (Pearson’s coefficient: *R* = 0.24 ± 0.05, *n* = 8) compared to the remainder of the cytosol (*R* = 0.38 ± 0.04, *n* = 8; **P < 0.01), measured using co-localization plugin JaCoP (ImageJ). A brightfield image is shown in *Figure 1A* (iv). Labelling of acidic organelles by LysoTracker™ in freshly isolated adult guinea pig atrial and ventricular myocytes revealed fluorescent acidic puncta throughout the cytosol (*Figure 1B*). In atrial myocytes, there was an additional concentration of acidic organelles at the nuclear poles, consistent with the published literature on the site of AG formation.^8^ Inhibition of PAM with PBA (200 µM), an essential component of AGs membranes,^8^ abolished LysoTracker™ labelling not only at the nuclear poles but the majority of acidic puncta in atrial myocytes (*Figure 1B*). In control conditions, atrial myocytes had a perinuclear (5 µm radius) acidic punctum count of 15.6 ± 1.2 per cell [*n* = 10; *Figure 1C* (i)] and a peripheral count of 10.7 ± .2.3 [*n* = 10; *Figure 1C* (ii)] at the top of the cell and 11.7 ± 1.4 [*n* = 10; *Figure 1C* (iii)] at the bottom of the cell. Addition of PBA caused a significant decrease in the number of punctum in both perinuclear and peripheral cytosol; the perinuclear acidic punctum count following PBA treatment was 4.4 ± 1.2 [*n* = 14, ****P < 0.0001; *Figure 1C* (i)] and peripheral count 3.5 ± 1.7 [*n* = 14, *P < 0.05; *Figure 1C* (ii)] at the top and 6.2 ± 1.9 [*n* = 14, *P < 0.05; *Figure 1C* (i)] at the bottom. Application of PBA to ventricular myocytes had no impact on LysoTracker™ labelling (*Figure 1D*). In control conditions, ventricular myocytes had a perinuclear acidic punctum count of 3.8 ± 0.6 and a peripheral count of 17.5 ± 1 1 at the top and 17.2 ± 3.2 at the bottom of the cell [*n* = 6; *Figure 1D* (i)–(iii)]. Following the addition of PBA, the perinuclear count was 5.2 ± 0.6 and peripheral punctum count was 12.8 ± 1.6 (top) and 13.7 ± 1.6 (bottom) in ventricular myocytes [*n* = 12, ns; *Figure 1D* (i)–(iii)].

### Atrial granule position changes significantly in a goat model of atrial fibrillation

**Figure 2:**
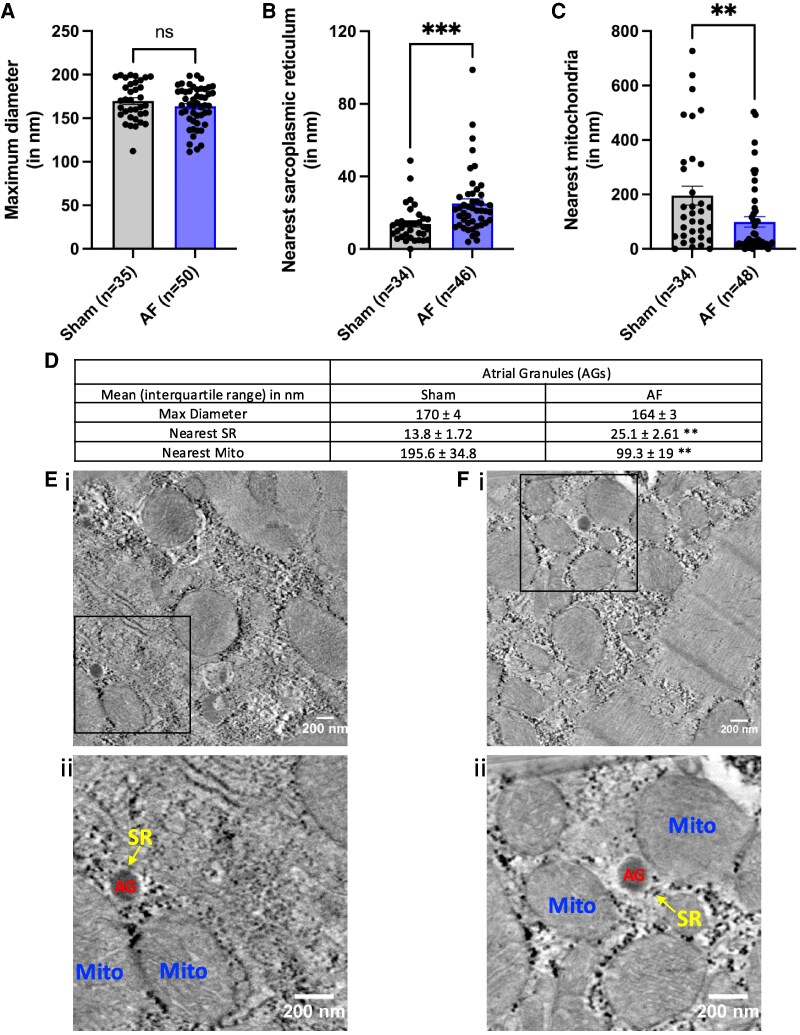
Atrial granule position changes significantly in a goat model of atrial fibrillation. (*A*) Maximum atrial granule diameter in sham-operated (*n* = 35) and atrial fibrillation goat (*n* = 50). (*B*) Minimum distance between atrial granules and nearest sarcoplasmic reticulum measured in sham-operated (*n* = 34) and atrial fibrillation goat tissue (*n* = 46). (*C*) Minimum distance between atrial granules and mitochondria in sham-operated (*n* = 34) and atrial fibrillation goats (*n* = 48). (*D*) Summary table indicating maximum diameter, nearest sarcoplasmic reticulum, and nearest mitochondria to atrial granules measured in sham-operated and atrial fibrillation goat tomograms. AGs, atrial granules; SR, sarcoplasmic reticulum; AF, atrial fibrillation. (*E*) 3D tomography snapshot (i) and the indicated zoomed-in area (square; ii) from the left atrium goat sample in sinus rhythm control. (*F*) 3D tomography snapshot (i) and the indicated zoomed-in area (square; ii) from the left atrium goat sample in atrial fibrillation. Snap shots in (*E*) and (*F*) show proximity of atrial granules (AG) to mitochondria (Mito) and sarcoplasmic reticulum (SR arrows). Scale bars provided on each image represent 200 nm.

We measured the size and position of AGs in relation to both the SR and mitochondria in left atrial tissue in a goat AF model using 3D electron tomography. Consistent with our live cell results, EM of fixed goat atrial tissue revealed the presence of both lysosomes and AGs in atrial myocytes. Atrial granules in sham-operated control samples had a maximum diameter of 170 ± 4 nm (*n* = 35, *n* = 4 goats; *Figure 2A*). In sham tissue, AGs were observed in close apposition to the SR (13.8 ± 1.72 nm at the closest point, *n* = 34, *n* = 4; *Figure 2B*) and were positioned at a minimum distance of 195.6 ± 34.8 nm (*n* = 34, *n* = 4) away from the nearest mitochondria (*Figure 2C*). After 6 months in AF, AGs showed no significant changes in diameter compared to those observed in sham-operated controls (maximum diameter 164 ± 3 nm, *n* = 50, *n* = 4 goats, *P* > 0.05; *Figure 2A*). In AF tissue, AGs were positioned significantly further away from their nearest SR, with closest distance of 25.1 ± 2.61 nm (*n* = 46, *n* = 4), compared to 13.8 ± 1.72 nm in sham-operated samples (***P < 0.001; *Figure 2B*). Atrial granules in AF goats were significantly closer to mitochondria than those in control cells, with the average closest distance of 99.3 ± 19 nm (*n* = 48, *n* = 4, **P < 0.01; *Figure 2C*). A summary table is included in *Figure 2D*, and representative snap shots from 3D electron tomograms of AGs (red text), mitochondria (Mito, blue), and SR (yellow arrows) are provided in *Figure 2E* (i) (sinus rhythm control) and *Figure 2F* (i) (AF) with zoomed in regions in *Figure 2E* (ii) and *Figure 2F* (ii), respectively.

## Discussion

Back in 1964, Jamieson and Palade^15^ described AGs as “large populations (up to 600/cell) of spherical, electron-opaque granules approximately 0.3 to 0.4 micro in diameter are characteristically found in muscle fibres of mammalian atria. They are absent in muscle fibres of the ventricles”. Because they contain acid phosphatase, AGs seem to be remnant bodies of autolytic foci.^15^ In this study, we have used PBA treatment to inhibit PAM, a protein present within AGs membranes, to demonstrate that AGs comprise a large percentage of acidic calcium stores within atrial cells. Here for the first time, we describe nanojunctions between AGs and the SR in mammalian cardiomyocytes. In goat AF samples, we observe significant structural remodelling between the positioning of AGs relative to the SR and mitochondria. We found that AGs are situated significantly further away from the SR and closer to mitochondria in goat AF tissue when compared to sham-operated controls (*Figure 2*). Similar observations have been made recently by our group regarding the positioning of lysosomes in relation to mitochondria and the SR.^9^ Our findings also emphasize the differences in acidic punctae between atrial and ventricular myocytes (*Figure 1*), and our ventricular experiments showed lower signal and no significant changes following PBA treatment, reinforcing the atrial-specific nature of AGs. Our immunofluorescence labelling highlights the lack of co-localization between AG-specific ANP and LAMP2, indicating that these compartments are largely distinct (*Figure 1A*). Morphological and enzyme cytochemical differences have been described previously between lysosomes and atrial-specific granules.^16^ The potentially strategic localization of AGs near the SR raises the question of whether functional cross talk exists between AGs and other calcium-containing organelles. LysoTracker™ imaging in *Figure 1* suggests a decrease in granule acidity. Furthermore, while the LysoTracker™ imaging experiments in *Figure 1* suggest a decrease in granule acidity in AGs following PBA treatment, they do not establish the complete disappearance of these organelles. It would be appropriate to note that the relatively short duration of PBA exposure (2 h) may be insufficient to fully eliminate granules, and thus, the observed effects likely reflect a functional alteration rather than physical removal. The lower levels of PAM in ventricular cells^7,8^ may account for the minimal effect of PBA in these cells, in contrast to its pronounced impact on atrial cells. Therefore, our results indicate that PBA disrupts AGs acidity, but cannot confirm whether the punctae remain in atrial myocytes after PBA treatment. Given their dual role in both calcium storage and hormone secretion, it will be important as a next step to explore whether AGs contribute to physiological calcium signalling in addition to their known hormone secretory role. As AF has been shown to cause significant structural remodelling at both the cellular and tissue level,^17^ further studies are needed to determine the physiological consequences of AG–SR–mitochondria interactions and how they may be altered in atrial pathophysiology.

## Data Availability

Data can be requested by contacting the lead contact. This study did not generate new unique codes.

## Lead author biography

Emily Akerman holds a Master’s in Biology and Medical Science (University of Bordeaux) and Bachelor’s in Life Science (University of Poitiers). From 2020 to 2024, she completed her PhD in Pharmacology at the Department of Pharmacology, University of Oxford, titled ‘Calcium signalling in atrial myocytes and potential targets for atrial fibrillation’, under the supervision of Prof. Rebecca Burton and Prof. Manuela Zaccolo. She recently started her first post-doctoral position at the Sainbiose Lab, University of Saint-Étienne, studying physiological changes in response to hypergravity and microgravity.
